# Improvement in Gait Abnormality Following Minimally Invasive Posterior Sacroiliac Joint Fusion

**DOI:** 10.7759/cureus.76853

**Published:** 2025-01-03

**Authors:** Nomen Azeem, Ajay Antony, Abhishek Kumar, Joel Verzosa, Sravani Thupili, Jon E Block

**Affiliations:** 1 Pain Management, Florida Spine and Pain Specialists, Riverview, USA; 2 Pain Management, The Orthopaedic Institute, Gainesville, USA; 3 Pain Medicine, The Orthopaedic Institute, Gainesville, USA; 4 Orthopaedics, Private Practice, San Francisco, USA

**Keywords:** allograft, a pilot study, gait, posterior fusion, sacroiliac joint fixation

## Abstract

Background

Sacroiliac joint (SIJ) dysfunction is a common cause of low back pain and associated gait disturbances that result from aberrant muscle activity and symmetry. This study evaluated the magnitude of improvement in gait characteristics in patients with chronic SIJ pain followed for six months after minimally invasive posterior SIJ fusion.

Methods

This was a single-arm, prospective, pilot study at two private practice orthopedic pain clinics. Gait characteristics were quantitated using a wireless wearable sensor. Ten patients (mean age: 63 ± 12 years) with abnormal SIJ-associated gait impairment were enrolled and underwent posterior SIJ fusion.

Results

Average gait velocity improved significantly from 69 ± 28.5 cm/sec at baseline to 99.9 ± 31.5 cm/sec at six months, reflecting an overall average improvement of 30.93 cm/sec or 55.4% (p=0.003). Gait speed, variability, and symmetry impairment parameters also improved with corresponding mean percentage improvements at six months of 27.8% (p=0.02), 19.7% (p=0.17), and 11% (p=0.27). A significant decrease in fall risk and increased timed-up-and-go assessments were noted, with improvements of 32.3% and 24.7%, respectively (p=0.004 for both comparisons).

Conclusion

These pilot findings demonstrate the first objective assessment of gait characteristics in patients with SIJ dysfunction undergoing minimally invasive posterior SIJ fusion.

## Introduction

Humans are the only species that ambulate bipedally with an upright or orthograde posture as their obligate form of locomotion [1]. In fact, the evolution of bipedalism was accompanied by significant evolutionary changes in spinal anatomy, resulting in the unique sinusoidal shape and flexibility of the vertebral column. This distinctive adaptation, however, increases the risk of osseo-ligamentous degeneration, particularly in the lumbosacral region of the spine [2,3].

A healthy gait pattern depends on an array of biomechanical features coordinated by the central nervous system to provide an economy of motion and stability [4]. Degenerative changes, injuries, and other pathologies can alter these features, resulting in marked gait deficits, often with detrimental consequences for energy expenditure and balance.

The spinopelvic complex serves as an important kinematic and load transfer junction during normal human gait [5,6]. As such, sacroiliac joint (SIJ) dysfunction is a common cause of low back pain and associated gait disturbances are a key clinical feature [7]. A number of studies have confirmed the association between SIJ dysfunction and gait impairment with aberrant muscle activity and symmetry identified as causative factors [8,9].

Quantitative measurement of gait is a valuable tool in spine care and rehabilitation [10]. This type of evaluation provides an assessment of each phase of the gait cycle as well as the variation over time and space. Wearable sensors are relatively low cost and can be used to accurately and reliably measure gait in clinical practice. We conducted a pilot study to evaluate the effect of minimally invasive, posterior SIJ fusion on quantitative gait parameters captured with wearable sensors in patients with gait impairment associated with SIJ dysfunction.

## Materials and methods

This was a prospective, single-arm, pilot study undertaken at two clinical sites in the US. The primary objective was to evaluate the magnitude of improvement in gait characteristics in patients with chronic SIJ pain followed for six months after minimally invasive, posterior SIJ fusion. As a feasibility investigation, this study was hypothesis-generating in exploring the therapeutic effectiveness of posterior SIJ fusion with an allograft implant to improve gait abnormalities [11]. Institutional Review Board (IRB) approval to conduct this trial was obtained on 3/31/22 from WIRB-Copernicus Group (WCG) (#20215866) and was conducted in accordance with the 1964 Helsinki Declaration. The first patient was enrolled on 7/25/22, and the last patient's final visit was on 12/6/23. Written informed consent was obtained from each study participant.

The number of enrolled participants was in accordance with sample size requirements for feasibility studies [11,12]. Patients were eligible for inclusion in this study if they were between 21 and 75 years of age and exhibited chronic low back, buttock, and/or leg pain of ≥6 months duration refractory to conservative care measures. Upon physical examination, all patients were required to show a positive response to at least three maneuvers specific to SIJ dysfunction (e.g., flexion, abduction, and external rotation (FABER), Gaenslen’s test, Stork/Gillet test, SIJ compression, and SIJ distraction) [13]. To confirm SIJ pain origin, eligible patients also needed to demonstrate ≥ 50% reduction in pain intensity following SIJ corticosteroid injection or lateral branch block. Imaging evidence of SIJ degeneration by standard radiography or computed tomography (CT) scan with or without a history of prior lumbar fusion was required. Study eligibility was restricted to patients with gait abnormality as measured by the Kinesis Gait™ analysis instrument (Kinesis Health Technologies, Dublin, Ireland).

All patients underwent minimally invasive, posterior SIJ fusion with allograft placement (LinQ, PainTEQ, Tampa, FL, USA). The details of this procedure have been published previously [14]. Briefly, under monitored anesthesia care, a small bone allograft implant is inserted through a small incision using specialized instruments into the SIJ and is designed to provide initial stabilization and fusion across the joint space.

Gait characteristics were measured using the Kinesis Gait™ monitor (Linus Health Europe Ltd., Dublin, Ireland), which is a portable clinical tool for quantitative assessment of gait and mobility. Using wireless sensors worn on the legs, Kinesis Gait™ monitors gait and mobility during a clinical walking test protocol with reference to a normative population [15-17]. Six gait domains were evaluated: gait velocity, speed impairment, variability impairment, symmetry impairment, fall risk, and timed-up-and-go (TUG) [18]. Impairment parameters were computed on a percentage scale (0-100%) in relation to a reference population of normal healthy individuals. For the TUG test, the patient was timed for getting up from a chair, walking three meters, turning at a designated spot, returning to the seat and sitting down. All gait parameters were measured at baseline and one and six months after the SIJ fusion procedure.

Findings are presented as mean (± standard deviation, SD) at baseline and each follow-up interval. Longitudinal improvements in gait parameters were computed as the mean (95% confidence interval, CI) raw change and average percentage (interquartile range, IQR) increase from baseline through six months. The difference between baseline values and the six-month endpoint was assessed using the paired t-test and two-tailed.

## Results

Ten patients (seven female, three male) with an average age of 63 ± 12 years met all inclusion and exclusion criteria and were enrolled as study subjects. Prior to fusion surgery, all patients demonstrated at least moderate gait impairment compared to normative values.

**Figure 1:**
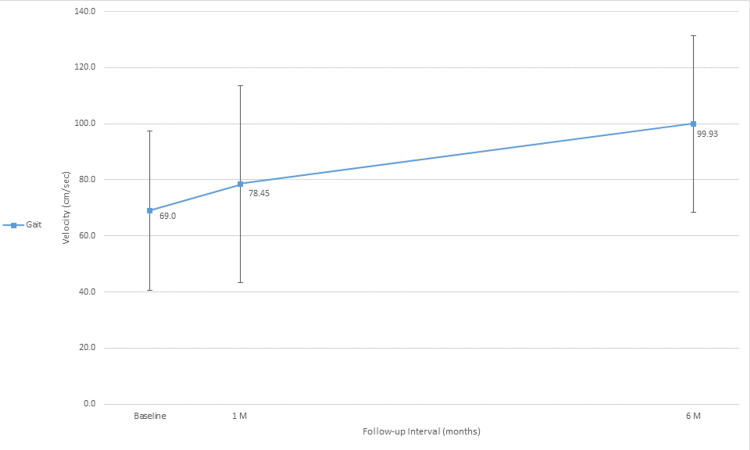
Line graph of gait velocity (cm/sec). Mean values for gait velocity at baseline, one and six months postoperatively. The overall average magnitude of improvement was 30.93 cm/sec, 95% CI (13.4, 48.4).

**Figure 2:**
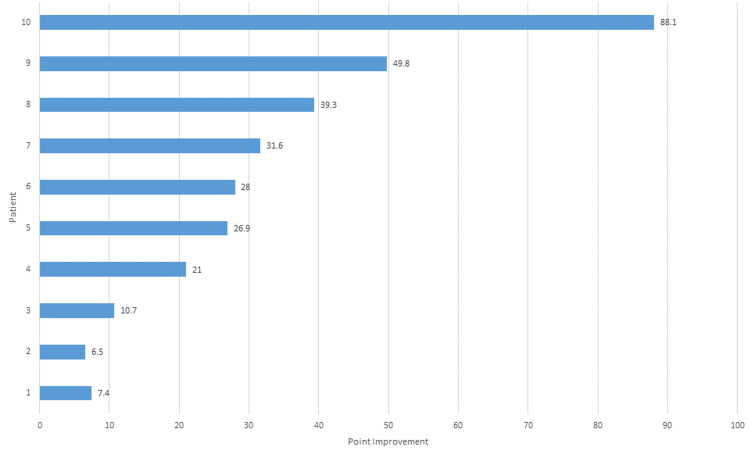
Distribution of gait velocity values. Gait velocity values were ranked by magnitude of improvement at six months over baseline for each patient.

Average gait velocity improved significantly from 69 ± 28.5 cm/sec at baseline to 78.5 ± 35 cm/sec and 99.9 ± 31.5 cm/sec at one and six months, respectively (Figure 1). The overall average magnitude of improvement was 30.93 cm/sec, 95% CI (13.4, 48.4), representing a mean percentage increase of 55.4% (IQR, 20% to 70%) (p=0.003). Figure 2 provides the individual raw change in gait velocity from baseline through six months of follow-up for each patient separately.

**Figure 3:**
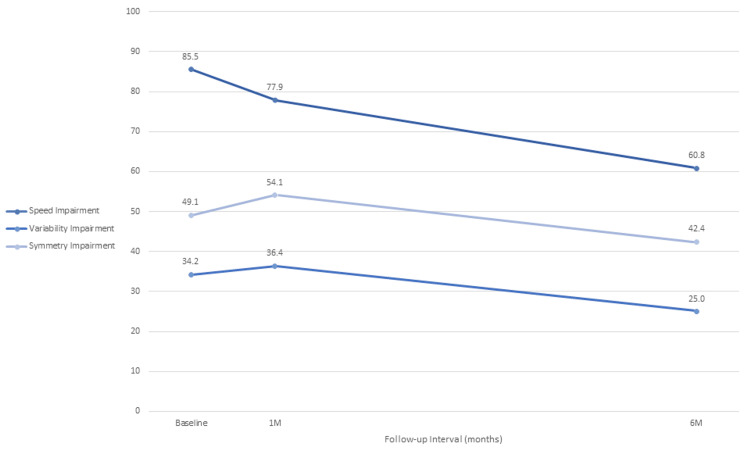
Line graph of gait speed impairment, variability impairment, and symmetry impairment. Gait speed impairment, variability impairment, and symmetry impairment at baseline, one and six months postoperatively. The overall average magnitudes of percentage point improvement for the three sub-domains were 24.7, 95% CI (5.7, 43.7); 9.2, 95% CI (-4.9, 23.3); and 6.7, 95% CI (-6.4, 19.8), respectively.

Post-operative improvements were also noted for the three impairment sub-domains. Figure 3 shows the mean values at baseline and follow-up intervals for gait speed, variability, and symmetry impairment. The overall average percentage point decreases were 24.7, 95% CI (5.7, 43.7), 9.2, 95% CI (-4.9, 23.3), and 6.7, 95% CI (-6.4, 19.8), respectively. Corresponding mean percentage improvements at six months over baseline were 27.8% (IQR: 7% to 46%) (p=0.02), 19.7% (IQR: -30% to 74%) (p=0.17), and 11% (IQR: 2% to 52%) (p=0.27).

The risk of falling decreased significantly from 50.7 ± 14.7 at baseline to 43.6 ± 14.3 and 34.6 ± 15.2 at one and six months, respectively, reflecting an overall average decrease of 16.11, 95% CI (7, 25.2). The corresponding percentage improvement at the final follow-up was 32.3% (IQR, 14% to 57.5%) (p=0.004).

Mean TUG values also decreased significantly from baseline (14.2 ± 6.5 secs) to one month (11.6 ± 4.5 secs) and six months (10.3 ± 4.0 secs), representing an overall average decrease of 3.85 secs, 95% CI (1.6, 6.1) or 24.7% (IQR, 18% to 28%) (p=0.004).

## Discussion

The results of this pilot study provide preliminary evidence that minimally invasive posterior SIJ fusion is associated with significant improvements in gait characteristics in patients with chronic SIJ dysfunction. Gait velocity (walking speed) is perhaps the most important measure of an individual’s gait. We found that the average gait velocity increased by approximately 55% in pilot subjects at six months following posterior SIJ fusion, and there was a corresponding 28% diminution in the severity of gait speed impairment.

The gait variability impairment parameter examines the variation in several quantitative gait parameters with high variability scores linked to the risk of falls [19]. We noted approximately 20% post-procedural improvement in this variable at six months, although the difference did not achieve statistical significance. Similarly, the gait symmetry impairment variable, which quantitatively assesses relationships in side-to-side stride patterns with high symmetry scores indicating gait asymmetry, also showed a non-significant longitudinal improvement of approximately 11% over baseline. We noted a slight increase in variability and symmetry impairment values one-month post-surgery (Figure 3), which may reflect a period of delayed recovery at this early follow-up interval.

Despite the small sample of patients enrolled in this pilot study, we also noted significant improvements in fall risk and TUG scores of approximately 32% and 25%, respectively, at the six-month clinical endpoint. Both of these variables provide a validated risk profile of a patient's future risk of having a fall [18,20]. Given the enormous costs associated with falls, a reduction in injury risk following posterior SIJ fusion could translate to substantial cost-utility benefits [21].

The results of this pilot study confirm and extend a previous report demonstrating improvement in gait characteristics in patients with SIJ dysfunction having lateral SIJ fusion [22]. Using an elaborate gait characteristic measurement protocol, that study also reported a significant increase in gait velocity through six months of post-procedural clinical follow-up. Our study, in contrast, utilized simple-to-use, wearable sensors that can be employed in a physician’s or physical therapy office setting to capture gait characteristics.

The limitations of this investigation include the small sample size and the relatively short duration of follow-up. It will be important to corroborate these feasibility findings in a larger population of patients with SIJ dysfunction and evaluate the extent of the durability of the treatment effect associated with posterior SIJ fusion in ameliorating SIJ-associated gait impairment [23,24].

## Conclusions

Posterior SIJ intra-articular fusion with an allograft implant has previously been shown to significantly improve pain and functional parameters. These pilot findings demonstrate the first objective assessment of gait characteristics in patients with SIJ dysfunction undergoing minimally invasive posterior SIJ fusion. Further research is encouraged to validate significant and durable improvements in gait impairment following posterior SIJ fusion.
